# Light Attention Encoder–Decoder for Cattle Body Segmentation and Body Weight Estimation

**DOI:** 10.3390/ani16121773

**Published:** 2026-06-08

**Authors:** Sahilpreet Singh Mann, Halah K. Shehada, Sabrina T. Amorim, Dong S. Ha, Gota Morota, Sook Shin

**Affiliations:** 1 Bradley Department of Electrical and Computer Engineering, Virginia Polytechnic Institute and State University, Blacksburg, VA 24061, USA; sahilpreetmann@vt.edu (S.S.M.); shehada@vt.edu (H.K.S.); dha@vt.edu (D.S.H.); 2 Department of Animal and Food Sciences, Oklahoma State University, Stillwater, OK 74078, USA; samorim@okstate.edu; 3 Laboratory of Biometry and Bioinformatics, Department of Agricultural and Environmental Biology, Graduate School of Agricultural and Life Sciences, The University of Tokyo, Bunkyo, Tokyo 113-8657, Japan; morota@ut-biomet.org

**Keywords:** boundary-aware segmentation, depth maps, gaussian context transformer, convolutional networks, U-Net

## Abstract

Accurate, non-invasive body weight estimation is essential for management and performance monitoring in beef cattle systems, yet conventional scales and manual measurements require animal handling, infrastructure, and labor. This study presents an integrated pipeline that segments cattle from overhead depth images and predicts body weight from extracted image features. The approach uses a Light Attention Encoder–Decoder (LAED) segmentation model combining depthwise separable convolutions, Gaussian Context Transformer (GCT) attention, a multi-scale dilated bottleneck, and dual heads for region and boundary prediction. Depth videos were collected using an overhead Intel RealSense D435 RGB-D camera from 60 beef heifers. To reduce animal-level leakage, leave-one-animal-out cross-validation was used for segmentation. LAED + GCT achieved 96.91% Dice (95% confidence interval (CI): 96.56–97.21%) and 94.22% IoU (95% CI: 93.58–94.77%), while operating at 33.08 frames per second. For weight prediction, biometric traits and deep features were evaluated using random forest, support vector regression, and fully connected neural networks. The best primary-metric body-weight model used biometric traits with support vector regression, achieving MAPE = 6.75%, pooled $R^{2}$ = 0.68, MAE = 23.92 kg, and RMSE = 31.79 kg. Among FCNN models trained independently within each cattle-level fold, the best result used ResNet50 features and achieved MAPE = 7.76%, a pooled $R^{2}$ = 0.56, an MAE = 27.60 kg, and an RMSE = 37.07 kg. The mean signed prediction bias for the biometric-SVR model was −1.04 kg, using predicted minus observed body weight, with a bootstrap 95% confidence interval of −9.63 to 7.41 kg. These results support the promise of overhead depth imaging for non-invasive cattle body segmentation and weight estimation, while larger external validation remains necessary.

## Simple Summary

Accurate body weight measurement is essential for animal evaluation, growth monitoring, and management decisions in beef cattle operations. However, conventional weighing systems require handling facilities, specialized equipment, and additional labor, which can increase operational costs and induce stress in animals. In this study, we developed an automated computer vision approach using an overhead depth camera to estimate cattle body weight non-invasively. Our system uses a new lightweight artificial intelligence model, the Light Attention Encoder–Decoder (LAED), to identify and segment cattle bodies in camera images, then calculate weight from body measurements. Testing on 60 beef cattle showed high animal-level segmentation accuracy with uncertainty estimates, real-time inference, and promising weight-prediction performance under the evaluation protocol used in this study. The best primary-metric body-weight model used automatically derived biometric traits with support vector regression, achieving 6.75% mean absolute percentage error, a pooled $R^{2}$ of 0.68, an MAE of 23.92 kg, and an RMSE of 31.79 kg. The mean signed prediction bias, defined as predicted minus observed body weight, was −1.04 kg, indicating slight average underestimation. However, the bootstrap analysis indicated that the bias was not significantly different from zero. This technology may offer farmers a cost-effective, non-invasive way to monitor cattle growth and make better management decisions.

## 1. Introduction

Body weight is a fundamental indicator of growth, nutritional status, and sexual maturity in beef cattle production systems. In replacement heifers, reaching an adequate target weight before breeding is essential to ensure sexual maturity, successful conception, and long-term productivity. Studies have shown that females calving at approximately 24 months of age achieve greater lifetime productivity, averaging 0.7 more calves and an additional 137.43 kg of weaned calf weight compared with animals that calve at 36 months [1]. Because feed accounts for approximately 60–70% of the total cost of heifer development, monitoring growth and body weight is critical for maintaining production efficiency. Achieving the recommended average daily gain of approximately 0.68 kg allows producers to reach breeding targets while avoiding excessive feeding costs or delayed reproductive performance.

Traditionally, body weight in cattle is measured using livestock scales [2] or estimated using weight tapes based on body measurements [3]. Although these methods are widely used, they require animal handling, dedicated infrastructure, and additional labor. Frequent handling can also increase animal stress and make routine weight monitoring difficult in commercial settings. These limitations highlight the need for alternative approaches that allow accurate, cost-effective, and non-invasive estimation of cattle body weight while minimizing animal disturbance. Automated weight monitoring may also support precision livestock management by enabling frequent phenotypic data collection without increasing animal handling or stress.

Advances in precision livestock farming have created new opportunities to monitor animal performance using automated sensing technologies. Image-based phenotyping systems, in particular, have received increasing attention as tools to collect objective measurements of livestock without the need for direct animal handling [4]. Within this context, computer vision approaches have been explored for estimating cattle body weight using digital images. These methods typically rely on either 2D RGB images, 3D depth images, or a combination of both modalities [5]. Depth-based imaging systems are particularly promising because they capture information on the distance between the animal and the camera, allowing accurate extraction of morphometric traits such as body height, length, and body volume. These features can then be incorporated into statistical or machine learning models to predict body weight [6].

Despite recent progress in computer-vision-based approaches for cattle weight estimation, reliable implementation under practical farm conditions remains challenging [7]. In commercial environments, top-down depth images frequently contain occlusions caused by animal movement, legs, rails, or handlers, as well as missing or noisy depth measurements. In addition, cattle vary substantially in body size, posture, and orientation relative to the camera, which can affect segmentation accuracy and the extraction of morphometric features used for weight prediction. Many existing approaches rely on manual or rule-based preprocessing steps or employ computationally intensive segmentation models that limit their suitability for real-time deployment [8]. Furthermore, cattle datasets are often relatively small and may include multiple observations from the same animals when growth-related traits are evaluated. Also, when model training and validation are not separated by animal identity, prediction performance may be artificially inflated due to data leakage [9]. These limitations highlight the need for segmentation approaches that are robust under practical farm conditions, computationally efficient, and evaluated using leakage-free validation strategies.

Recent studies have expanded automated cattle weight-estimation methods using different sensors and modeling strategies. Ruchay et al. [10] combined RGB-D morphometric traits with movement-speed features from a multi-camera Kinect v2 system and YOLOv8n detection. Nilchuen et al. [11] proposed a smartphone-based YOLOv11m system for side-view Brahman cattle measurements, while Menezes et al. [12] used top-down Intel RealSense D435 videos with keypoint and monocular–depth-estimation features for body weight and hip height prediction. These studies demonstrate important progress but also highlight continuing trade-offs among hardware complexity, sensor cost, segmentation granularity, computational efficiency, and validation design.

To address these challenges, the objective of this study was to develop and evaluate a computer-vision pipeline for automated cattle body segmentation and body weight estimation using overhead depth imagery. Specifically, we propose a Light Attention Encoder–Decoder (LAED) segmentation architecture designed to improve robustness under practical farm conditions, where occlusion, variation in body size, and differences in animal posture can affect image-based measurements. The proposed model integrates Gaussian Context Transformer (GCT) attention modules and multi-scale dilated convolutions within the network bottleneck to better capture variation in cattle body shape while maintaining computational efficiency. The architecture also incorporates joint semantic segmentation and boundary-aware learning to improve the delineation of cattle body contours. The proposed approach was evaluated using animal-disjoint validation, uncertainty estimation, paired statistical comparisons, computational-efficiency metrics, and downstream body weight prediction from biometric descriptors and deep image features. By improving the accuracy, robustness, and efficiency of automated phenotyping from depth imagery, this framework has the potential to support precision livestock farming applications and facilitate scalable, non-invasive monitoring of cattle growth and performance under commercial production conditions.

## 2. Materials and Methods

### 2.1. Animals and Ethics

Animal handling and data collection procedures were approved by the Virginia Tech Institutional Animal Care and Use Committee (IACUC protocol code: 22-137). The study included 60 beef heifers from the Virginia Tech Beef Center (Blacksburg, VA, USA), each approximately 12 months of age. The animals in this study were represented by Angus, Charolais, and Hereford, and had an average body weight of 351.27 kg, with a standard deviation of 54.32 kg. The median recorded body weight was 369.2 kg, the range was 221.8–562.5 kg, and the Inter Quartile Range (IQR) was 63.72. The heifers were maintained in paddocks under standard management conditions, with ad libitum access to feed and water.

### 2.2. Data Collection

Data collection was conducted between September 2022 and April 2023 at the Virginia Tech Beef. During each data collection session, heifers were individually guided through a one-way lane located between the entry and exit points of the working chute. An Intel RealSense D435 RGB-D camera (Intel Corporation, Santa Clara, CA, USA) was mounted approximately 2.5 m above the ground to capture synchronized RGB and depth data from a top-down perspective. RGB-D cameras estimate depth by combining information from multiple image sensors to calculate the distance between the camera and objects in the scene [13].

**Figure 1:**
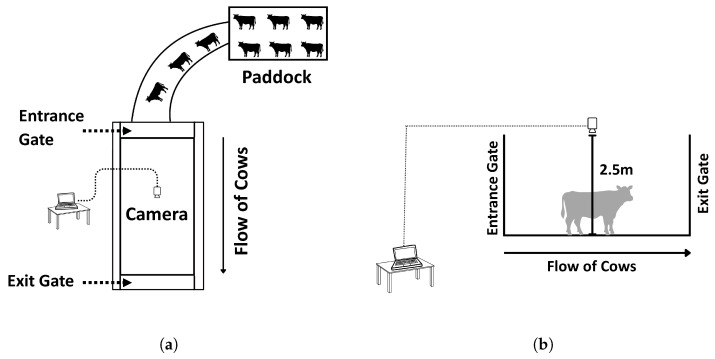
Illustration of the data acquisition setup for top-view depth imaging of beef cattle using an overhead RGB-D camera. The camera is mounted above a one-way lane, capturing depth data as individual animals pass beneath it. (**a**) Top-view schematic of the cattle depth imaging setup, showing the overhead RGB-D camera capturing depth data as animals pass through the pen to record weight in line. (**b**) Side-view schematic illustrating the height (2.5 m) and positioning of the RGB-D camera relative to the cattle passage lane.

Approximately 30 s of video were recorded for each animal at 15 frames per second as the heifers moved through the lane. The overhead camera configuration ensured that only one animal was captured at a time, minimizing occlusion and allowing clear visualization of the dorsal body surface. Figure 1 illustrates the top-view layout and side-view schematic of the data collection setup.

The depth-sensing camera was connected to a laptop computer via a USB 3.0 interface and controlled using Intel RealSense software v2.58.1. Videos were recorded in the proprietary .bag format and subsequently converted into RGB image files (PNG format) and corresponding depth matrices (CSV format) for further processing and analysis.

### 2.3. Dataset Annotation and Preprocessing

A labeled dataset of 60 depth frames was constructed, consisting of one manually selected frame per animal (i.e., one image per cattle ID). The selected frames were taken from the top-down video sequence and used for pixel-wise annotation. Pixel-wise ground-truth segmentation masks were created using the LabelMe annotation tool v6.3.1 [14].

Prior to network input, depth images were normalized using a percentile-based procedure to reduce sensitivity to extreme values and sensor artifacts. Specifically, the 1st and 99th percentiles were used as lower and upper bounds, and depth values were clipped to this interval to obtain $D_{\mathrm{clip}}$. The clipped image was then linearly mapped to [−1,1]:

(1) $$D_{\mathrm{norm}}=2\times \frac{D_{\mathrm{clip}}-D_{\min}}{D_{\max}-D_{\min}}-1,$$

where $D_{\min}$ and $D_{\max}$ denote the minimum and maximum values after clipping. This scaling improves robustness to variable operating ranges and depth noise.

For the auxiliary boundary supervision, a boundary target is derived from each ground-truth mask by applying morphological erosion with a 3×3 kernel and subtracting the eroded mask from the original mask.

### 2.4. Proposed LAED Segmentation Architecture

To accurately segment cattle bodies from overhead depth imagery under substantial pose and scale variation, we propose LAED, a lightweight encoder–decoder network with Gaussian Context Transformer (GCT) [15] attention. Our design is inspired by the shape- and boundary-aware modeling motivation of SAUNet [16], but differs substantially in architecture and application. In particular, SAUNet employs a dual-stream texture/shape framework with a gated shape stream for medical image segmentation, whereas LAED uses a single-stream backbone with depthwise separable convolutions, multi-scale dilated bottlenecking, and an auxiliary boundary head tailored for real-time cattle segmentation.

LAED consists of a dual-head encoder–decoder architecture optimized for cattle segmentation from depth imagery. The network is designed to process single-channel depth maps and generate both semantic segmentation masks and boundary predictions simultaneously. Building upon established U-Net principles, the proposed segmentation model incorporates several key innovations: (1) GCT attention module at multiple encoder stages [15], (2) multi-scale dilated convolutions [17] in the bottleneck layer, and (3) dual prediction heads for joint segmentation and boundary detection.

The architecture leverages separable convolutions throughout the network to reduce computational complexity while maintaining feature extraction capability. Group Normalization [18] was employed instead of Batch Normalization [19] to ensure stable training with small batch sizes. The SiLU (Sigmoid Linear Unit) activation function [20] was utilized throughout the network for its smooth gradient properties and improved convergence characteristics.

**Figure 2:**
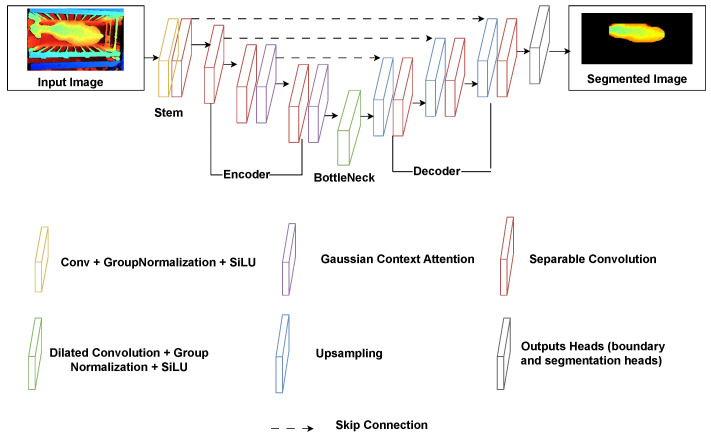
Architecture of the proposed LAED + GCT for cattle body segmentation from depth imagery. The network follows a lightweight U-Net encoder–decoder design with a stem feature extractor, separable convolution blocks for efficient representation learning, and GCT attention modules (purple) to incorporate global context at deeper encoder stages. A multi-scale dilated bottleneck (green) captures wider receptive fields without additional downsampling. The decoder progressively upsamples features (blue) and fuses them with encoder features through skip connections (dashed) to recover fine boundary details. Final dual output heads (gray) produce the segmentation mask and an auxiliary boundary prediction for improved edge localization.

Figure 2 illustrates the complete architecture of LAED, showing the information flow from input depth map through the encoder stages, bottleneck processing, decoder stages with skip connections, and finally to the dual prediction heads.

#### 2.4.1. Stem Layer

The network begins with a stem layer designed to extract initial low-level features from the input depth map. The stem consists of two primary components arranged sequentially. First, a standard 7×7 convolution with a stride of 1 and a padding of 3 processes the single-channel input, expanding it to the base channel dimension. This large receptive field enables the network to capture coarse spatial patterns. The convolution is followed by Group Normalization and SiLU activation. Second, a separable convolution block refines these initial features while maintaining the same spatial resolution.

The stem layer can be expressed mathematically as:

(2) $$F_{\mathrm{stem}}=\mathrm{SepConv}\left(\mathrm{SiLU}\left(\mathrm{GN}\left({\mathrm{Conv}}_{7\times 7}\left(I_{\mathrm{depth}}\right)\right)\right)\right)$$

where $I_{\mathrm{depth}}$ represents the input depth map and $F_{\mathrm{stem}}$ denotes the stem output features.

#### 2.4.2. Separable Convolution Block

The separable convolution block (SepConv2d) module implements depthwise separable convolutions, which factorize standard convolutions into two sequential operations: depthwise convolution followed by pointwise convolution. This factorization reduces computational cost from $k^{2}\times C_{\mathrm{in}}\times C_{\mathrm{out}}$ to $k^{2}\times C_{\mathrm{in}}+C_{\mathrm{in}}\times C_{\mathrm{out}}$, where $C_{\mathrm{in}}$ and $C_{\mathrm{out}}$ are input and output channels, respectively. Each SepConv2d block follows the following sequence:

(3) $$F_{\mathrm{out}}=\mathrm{SiLU}\left(\mathrm{GN}\left({\mathrm{Conv}}_{1\times 1}\left({\mathrm{Conv}}_{k\times k}^{\mathrm{DW}}\left(F_{\mathrm{in}}\right)\right)\right)\right)$$

where ${\mathrm{Conv}}_{k\times k}^{\mathrm{DW}}$ indicates depthwise convolution and ${\mathrm{Conv}}_{1\times 1}$ represents pointwise convolution.

#### 2.4.3. Gaussian Context Transformer

GCT was used as a lightweight channel-attention block. In contrast to spatial self-attention modules that compute pairwise interactions across spatial positions, GCT aggregates global context via global average pooling and produces channel-wise gates through a Gaussian excitation. Given a feature map $F_{\mathrm{enc}}\in R^{C\times H\times W}$, GCT first computes channel-wise global contexts using global average pooling:

(4) $$z=\mathrm{GAP}\;\left(F_{\mathrm{enc}}\right)\in R^{C}.$$

A Gaussian excitation produces channel-wise attention activations:

(5) $$a=\exp\;\left(-\frac{{\hat{z}}^{⊙2}}{2c^{2}}\right),$$

where $\hat{z}$ is obtained by zero-mean unit-variance normalization of *z* and ${\hat{z}}^{⊙2}$ denotes element-wise squaring. The attention is applied by broadcast element-wise multiplication:

(6) $$F_{\mathrm{att}}=\mathrm{GCT}\;\left(F_{\mathrm{enc}}\right)=F_{\mathrm{enc}}⊙a,$$

where ⊙ denotes element-wise multiplication with channel-wise broadcasting and *c* is the standard deviation.

#### 2.4.4. Encoder Path

The encoder progressively downsamples the feature maps while increasing channel depth, following a hierarchical feature extraction strategy. The encoder consists of four stages, each reducing spatial resolution by half while expanding the feature channels by a factor of two. The GCT attention modules at stages 3 and 4 enable the network to focus on salient features at medium and high semantic levels.

#### 2.4.5. Multi-Scale Dilated Bottleneck

The bottleneck layer processes the deepest features using parallel dilated convolutions to capture multi-scale contextual information without spatial downsampling. Following the convolutional bottleneck refinement, three parallel 3×3 convolutions with dilation rates of 1, 2, and 4 extract features at different receptive field scales. These multi-scale features were summed element-wise, normalized via Group Normalization, and activated with SiLU.

#### 2.4.6. Decoder Path with Skip Connections

The decoder reconstructs spatial resolution through a series of upsampling blocks, each combining transposed convolution with skip connections from corresponding encoder stages. The U-Net-style skip connections concatenate encoder features with upsampled decoder features, enabling the decoder to recover fine-grained spatial details lost during downsampling.

#### 2.4.7. Dual Prediction Heads

LAED employs two parallel prediction heads operating on the final decoder features to generate complementary outputs:

- Segmentation Head: A 1×1 convolution projects features to the number of classes (binary segmentation: background and foreground), producing logits of shape [B,2,H,W] where *B* is the batch size, *H* and *W* are the spatial dimensions. Cross-entropy loss is computed on these logits during training.
- Boundary Head: A separate 1×1 convolution generates single-channel boundary predictions of shape [B,1,H,W]. Binary cross-entropy with logits loss is applied to this head.

The dual-head design enables the network to learn both region-level segmentation and boundary-level refinement simultaneously.

#### 2.4.8. Alternative Attention Modules for Comparative Evaluation

To determine whether the performance gain of the proposed LAED model was specific to the Gaussian Context Transformer (GCT), three established attention mechanisms were also evaluated as alternative attention modules within the same LAED framework: Convolutional Block Attention Module (CBAM) [21], Dual Attention Network (DualAttn) [22], and Efficient Channel Attention (ECA) [23]. In these comparative experiments, the selected attention module was substituted for GCT while the remaining encoder–decoder architecture was kept unchanged.

Convolutional Block Attention Module (CBAM) is a lightweight attention mechanism that refines intermediate feature maps sequentially in the channel and spatial dimensions. The channel attention branch aggregates global context using both average pooling and max pooling across spatial locations, followed by a shared multilayer perceptron and sigmoid activation to generate channel-wise attention weights. The spatial attention branch then applies average pooling and max pooling across channels, concatenates the resulting two-dimensional maps, and estimates a spatial attention map using a 7×7 convolution followed by a sigmoid function. The input feature map is therefore first recalibrated along the channel dimension and then refined spatially.

Dual Attention Network (DualAttn) models long-range dependencies in two complementary domains: spatial position and channel. The position attention module computes pairwise relationships between spatial locations so that each position can be updated as a weighted sum of features from all positions, thereby incorporating global contextual information. In parallel, the channel attention module captures interdependencies among feature channels by computing attention weights across channels and refining each channel using information from the full set of channel responses. The outputs of the two branches are then fused to strengthen feature representation and improve segmentation consistency.

Efficient Channel Attention (ECA) is a lightweight channel-attention mechanism designed to avoid the dimensionality reduction commonly used in squeeze-and-excitation-style modules. After global average pooling compresses the spatial dimensions, local cross-channel interactions are modeled using a one-dimensional convolution. The convolution kernel size can be adaptively selected as a function of channel dimension, allowing efficient modeling of neighboring channel dependencies with very limited parameter overhead. The resulting channel weights are passed through a sigmoid function and applied to the feature map by channel-wise multiplication.

### 2.5. Training Strategy

#### 2.5.1. Data Augmentation

To improve generalization under limited supervision, geometric augmentation was applied during training. Horizontal flipping was performed with a probability of 0.5 and applied consistently to both the depth image and its corresponding mask.

#### 2.5.2. Loss Function

Training minimizes a composite objective that couples region classification with boundary localization:

(7) $$L_{\mathrm{total}}=L_{\mathrm{seg}}+L_{\mathrm{bound}}.$$

The segmentation term $L_{\mathrm{seg}}$ is the pixel-wise multi-class cross-entropy loss:

(8) $$L_{\mathrm{seg}}=-\frac{1}{N}\sum_{i=1}^{N}\sum_{c=1}^{C}y_{i,c}\log\left({\hat{y}}_{i,c}\right),$$

where *N* is the number of pixels, *C* is the number of classes, $y_{i,c}$ denotes the ground-truth indicator, and ${\hat{y}}_{i,c}$ is the predicted class probability.

The boundary term $L_{\mathrm{bound}}$ is implemented using binary cross-entropy with logits:

(9) $$L_{\mathrm{bound}}=-\frac{1}{N}\sum_{i=1}^{N}\left[b_{i}\log\left(\sigma \left({\hat{b}}_{i}\right)\right)+\left(1-b_{i}\right)\log\left(1-\sigma \left({\hat{b}}_{i}\right)\right)\right],$$

where $b_{i}$ is the boundary label, ${\hat{b}}_{i}$ is the predicted boundary logit, and σ(·) denotes the sigmoid function.

#### 2.5.3. Optimization

Parameters were optimized with AdamW [24] using an initial learning rate of $3\times 10^{-4}$ and weight decay of $1\times 10^{-4}$. Training was conducted for 10 epochs per fold with a batch size of 4. Given the small batch regime, Group Normalization was used in place of Batch Normalization to avoid unstable batch-statistic estimates.

### 2.6. Leave-One-Cattle-Out Cross-Validation for Segmentation

To maximize utilization of the limited labeled set, leave-one-cattle-out cross-validation (LOAO-CV) was adopted. For a dataset of *N* cattle, LOAO-CV comprises *N* folds; in fold *i*, cattle *i* is held out for testing while the remaining N−1 samples are used for training. In each fold, the model was re-initialized, trained for a fixed number of epochs, and evaluated on the held-out animal. This protocol provides per-animal predictions across the full cohort and supports an approximately unbiased estimate of generalization performance in the small-sample setting.

Dice and IoU were computed for each held-out animal and then averaged across animals. Therefore, the reported segmentation metrics were based on animal-level scores rather than pooling all pixels across the complete dataset into one confusion matrix. Segmentation uncertainty was summarized using mean ± standard deviation and nonparametric bootstrap 95% confidence intervals across animals. Because the same animals were evaluated across attention variants, paired MaxT permutation tests were used to compare LAED + GCT with the alternative attention modules and ablation variants. All uncertainty estimates and paired statistical comparisons for segmentation were computed at the animal level. Thus, the animal ID, rather than the pixel or frame, was used as the resampling and permutation unit.

### 2.7. Statistical Analysis for Segmentation Model Comparisons

Segmentation uncertainty was summarized using mean ± standard deviation and nonparametric bootstrap 95% confidence intervals. Bootstrap confidence intervals were computed using 20,000 bootstrap resamples with replacement at the animal level. In each bootstrap resample, cattle IDs were sampled with replacement, and the mean Dice or IoU was recalculated from the resampled animal-level scores. The reported 95% confidence intervals are percentile bootstrap intervals based on the 2.5th and 97.5th percentiles of the bootstrap distribution.

Because the same held-out animals were evaluated across segmentation model variants, paired statistical tests were performed using animal-level paired differences. The primary pairwise comparison used a two-sided studentized MaxT paired permutation test. For each metric, LAED + GCT was compared with each comparator model using paired animal-level differences. Under the null hypothesis of no paired difference, the signs of the animal-level paired differences were randomly flipped. For each permutation, studentized paired test statistics were computed for all pairwise contrasts within the metric, and the maximum absolute statistic across contrasts was recorded. The two-sided MaxT-adjusted *p*-value for each contrast was calculated as the proportion of permutations in which the maximum absolute permuted statistic was greater than or equal to the observed absolute statistic for that contrast.

For attention-module comparisons, 20,000 animal-level sign-flip permutations were used. The reported *p*-values for attention-module comparisons are two-sided MaxT-adjusted *p*-values and control the family-wise error rate across the four pairwise comparisons within each metric. Dice and IoU were treated as separate test families. For ablation analyses, the same animal-level paired MaxT permutation procedure was used, with full LAED + GCT as the reference model. MaxT adjustment was applied separately within Dice and IoU across the ablation contrasts.

### 2.8. Cattle Body Weight Estimation

For each animal, body weight was measured at the time of data acquisition. Using the predicted segmentation masks, automatically derived biometric features and deep features were extracted from each segmented image. The biometric descriptors were derived from segmented depth images and were not manually measured. These descriptors included foreground area, minimum-area-rectangle body dimensions, centroid coordinates, orientation, and an ellipsoid volume proxy:

(10) $$V_{\mathrm{proxy}}=\frac{4}{3}\pi \left(\frac{L}{2}\right)\left(\frac{W}{2}\right)\left(\frac{H}{2}\right),$$

where *L*, *W*, and *H* denote estimated length, width, and height, respectively. Deep features were extracted using DenseNet121 [25], ResNet50 [26], and ResNeXt50 [27]. To reduce the effect of outliers, each feature was aggregated per animal by taking the median across the segmented frames, resulting in one feature vector per cattle ID.

Body weight prediction was evaluated using 20-fold cross-validation at the cattle level, with three cattle held out for testing in each fold and the remaining cattle used for training. This grouping ensured that all observations from a given animal remained within a single fold and prevented animal-level leakage between training and testing. The 20 outer test folds were fixed before model fitting and included 60 unique cattle, with each animal appearing exactly once as a held-out test case. The fixed outer test folds were used only for final out-of-fold prediction and were not used for feature scaling, dimensionality reduction, hyperparameter tuning, early stopping, model selection, or final model fitting. All preprocessing steps, hyperparameter selection, and regression model fitting were performed exclusively within each training fold and then applied to the corresponding held-out cattle. Performance was computed from pooled out-of-fold predictions across all held-out cattle. In particular, $R^{2}$ was not computed separately within each three-animal fold and then averaged. Performance was reported using animal-level mean absolute percentage error (MAPE), coefficient of determination ($R^{2}$), mean absolute error (MAE), and root mean squared error (RMSE).

For Support Vector Regression (SVR), hyperparameters were selected only within the corresponding outer-training cattle. The three held-out cattle in the outer test fold were not used for SVR hyperparameter tuning, feature scaling, model selection, or final model fitting. After hyperparameter selection, the final SVR model for that fold was fit using only the outer-training cattle and then applied once to the held-out cattle.

For Fully Connected Neural Network (FCNN) models, each outer fold was trained independently. For each fold and ensemble member, the FCNN and optimizer were initialized from scratch. No model weights, optimizer states, fitted feature scalers, principal component analysis transformations, target-standardization parameters, validation information, or other training information were transferred across folds. Within each outer training fold, an inner validation split was created using only outer-training cattle and was used only for early stopping. After selecting the best epoch from the inner validation split, the final FCNN was retrained from scratch on all outer-training cattle and applied to the held-out cattle. Three independent FCNN ensemble members were trained per fold, and their predictions were averaged.

The FCNN used two hidden layers with 64 and 32 units, ReLU activation, Layer Normalization, dropout of 0.35, AdamW optimization with learning rate $1\times 10^{-3}$ and weight decay $1\times 10^{-3}$, Smooth L1 loss, gradient clipping at 5.0, target standardization, and PCA with up to 20 components fit only on training cattle. The maximum number of inner-training epochs was 700, with early-stopping patience of 80 epochs.

For the best-performing body-weight model, signed prediction bias was also computed from pooled out-of-fold predictions. Signed error was defined as predicted minus observed body weight:

(11) $$e_{i}={\hat{y}}_{i}-y_{i},$$

where positive values indicate overestimation and negative values indicate underestimation. Mean prediction bias was calculated as

(12) $$\mathrm{Bias}=\frac{1}{n}\sum_{i=1}^{n}e_{i}.$$

A nonparametric bootstrap 95% confidence interval for bias was computed by resampling cattle-level out-of-fold predictions.

### 2.9. Computational Efficiency Evaluation

To assess suitability for practical deployment, computational efficiency was evaluated on a Google Colab runtime equipped with an NVIDIA L4 GPU and 12.7 GB of host memory. Inference was benchmarked using a batch size of 1 on a single normalized depth image. To obtain stable timing estimates, 30 warm-up iterations were performed, followed by 300 timed forward passes. CUDA operations were synchronized immediately before and after the timed loop to ensure accurate GPU latency measurement. Mean latency (ms/image), throughput (frames/s), number of parameters, floating-point operations (GFLOPs), peak GPU memory, peak host RAM, and average CPU utilization were recorded during inference.

### 2.10. Performance Metrics

The proposed segmentation models were evaluated using mean Dice coefficient (mDice), mean intersection over union (mIoU), frames per second (FPS), parameters, and GFLOPs. mDice and mIoU quantify the overlap between predicted and ground-truth masks, thereby assessing how accurately the cattle body is segmented for downstream feature extraction. FPS, parameter count, and GFLOPs measure computational efficiency and indicate whether the model is suitable for real-time on-farm deployment. Body weight prediction was evaluated using MAPE, $R^{2}$, MAE, and RMSE:

(13) $$\begin{array}{cc} \mathrm{Dice} & =\frac{2TP}{2TP+FP+FN}, \end{array}$$

(14) $$\begin{array}{cc} \mathrm{IoU} & =\frac{TP}{TP+FP+FN}, \end{array}$$

(15) $$\begin{array}{cc} \mathrm{MAPE} & =\frac{100}{n}\sum_{i=1}^{n}\left|\frac{{\hat{y}}_{i}-y_{i}}{y_{i}}\right|. \end{array}$$

(16) $$\mathrm{MAE}=\frac{1}{n}\sum_{i=1}^{n}\left|{\hat{y}}_{i}-y_{i}\right|$$

(17) $$\mathrm{RMSE}=\sqrt{\frac{1}{n}\sum_{i=1}^{n}{\left({\hat{y}}_{i}-y_{i}\right)}^{2}}$$

The pooled coefficient of determination was computed as

(18) $$R^{2}=1-\frac{\sum_{i=1}^{n}{\left(y_{i}-{\hat{y}}_{i}\right)}^{2}}{\sum_{i=1}^{n}{\left(y_{i}-\bar{y}\right)}^{2}}.$$

Prediction bias was computed as the mean signed error:

(19) $$e_{i}={\hat{y}}_{i}-y_{i},\;\mathrm{Bias}=\frac{1}{n}\sum_{i=1}^{n}e_{i}.$$

where TP stands for true positives, FN stands for false negatives, FP stands for false positives, $y_{i}$ is the observed body weight, and ${\hat{y}}_{i}$ is the predicted body weight. For signed error, positive values indicate overestimation and negative values indicate underestimation because the sign convention was predicted minus observed body weight.

## 3. Results

### 3.1. Segmentation Performance

The proposed LAED with GCT attention module achieved the highest observed segmentation performance among the evaluated attention variants. Table 1 reports animal-level mDice and mIoU with mean ± standard deviation and bootstrap 95% confidence intervals. LAED + GCT achieved 96.91% mDice and 94.22% mIoU. Compared with LAED without attention and with DualAttn, ECA, or CBAM attention, LAED + GCT showed the highest mean Dice and IoU values.

Because the same animals were used to evaluate the attention variants, paired MaxT permutation tests were conducted using animal-level scores. The *p*-values in Table 2 are two-sided studentized MaxT-adjusted paired permutation *p*-values. Adjustment was performed separately for Dice and IoU across the four pairwise comparisons with LAED + GCT. As shown in Table 2, LAED + GCT showed statistically significant improvements over CBAM and DualAttn, while differences relative to no attention and ECA were not significant in the present sample of 60 animals.

Table 3 shows the metrics of the proposed model against baseline segmentation models. The proposed model achieved higher observed mDice and mIoU than U-Net, SAM2-U-Net, and Mask R-CNN while also requiring fewer parameters and lower GFLOPs. Mask R-CNN was retained as a widely used reference segmentation model, although its instance-segmentation objective is not identical to the single-object semantic segmentation task considered in this study.

To quantify the contributions of the boundary head and multi-scale dilated bottleneck, additional ablation experiments were conducted. Ablation *p*-values were computed using the same animal-level paired MaxT permutation framework, with full LAED + GCT as the reference model. MaxT adjustment was applied separately for Dice and IoU across the ablation contrasts. As shown in Table 4, removing the boundary head caused a small, non-significant reduction in segmentation performance. Removing the dilated bottleneck caused a larger decrease in mDice and mIoU, indicating that the multi-scale dilated bottleneck was the main architectural contributor under the present experimental setting.

### 3.2. Computational Efficiency

To quantify deployment efficiency, inference of the proposed LAED + GCT model was benchmarked on a Google Colab runtime with an NVIDIA L4 GPU using a batch size of 1. The model achieved 7.03 M parameters, 117.41 GFLOPs, a mean latency of 30.23 ms per image, and 33.08 FPS. Because the depth videos were acquired at 15 FPS, the proposed model operated at approximately 2.2 times the camera acquisition rate, indicating that segmentation can be performed in real time without becoming the throughput bottleneck of the image acquisition pipeline.

### 3.3. Body Weight Estimation Performance

Cattle body weight was estimated by extracting features from segmented images using ResNet50, DenseNet121, ResNeXt50, as well as automatically derived biometric traits. These features were subsequently provided to Random Forest (RF), SVR, and FCNN for body weight prediction. Body weight estimation was evaluated using 20-fold cross-validation at the cattle level, with three cattle held out for testing in each fold. Final performance was computed from pooled out-of-fold predictions across all held-out cattle.

**Figure 3:**
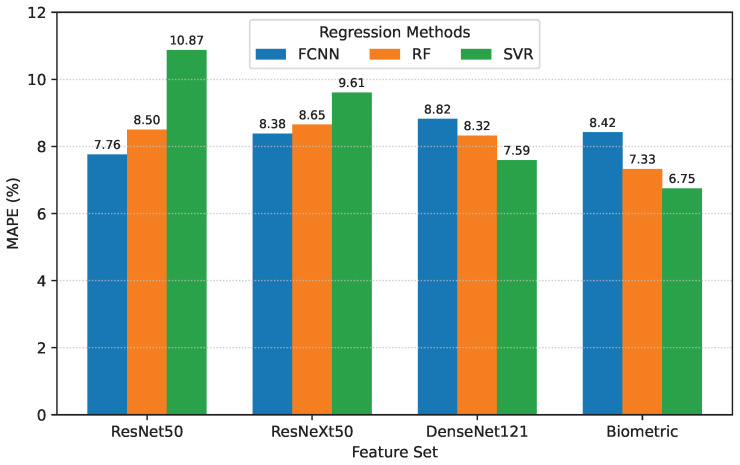
Mean absolute percentage error (MAPE, %) for cattle body weight estimation using FCNN, RF, and SVR across four feature sets (Biometric, DenseNet121, ResNet50, and ResNeXt50). The lowest MAPE was obtained using biometric traits with SVR. For this model, signed prediction bias was −1.04 kg, using predicted minus observed body weight. The best FCNN result was obtained using ResNet50 features.

As shown in Table 5 and Figure 3, prediction performance varied across feature sets and regression algorithms. Using MAPE and pooled $R^{2}$ as the primary evaluation criteria, the best model was SVR with biometric traits. This model achieved MAPE = 6.75%, pooled $R^{2}$ = 0.68, MAE = 23.92 kg, and RMSE = 31.79 kg.

Prediction bias was evaluated for this biometric-SVR model using signed error, defined as predicted minus observed body weight. The pooled held-out mean bias was −1.04 kg, indicating slight average underestimation. The bootstrap 95% confidence interval for bias was −9.63 to 7.41 kg, and the median signed error was 0.01 kg. Because the confidence interval included zero, the pooled out-of-fold predictions did not show strong evidence of systematic overestimation or underestimation. The signed-error standard deviation was 33.85 kg, indicating that individual-animal prediction errors remained variable even though the average bias was small.

Among the FCNN models trained with independent fold-specific initialization, the best result was obtained with ResNet50 features. This configuration achieved RMSE = 37.07 kg, MAE = 27.60 kg, pooled $R^{2}$ = 0.56, and MAPE = 7.76%. FCNN models with ResNeXt50, biometric, and DenseNet121 features achieved MAPE values of 8.38%, 8.42%, and 8.82%, respectively.

Biometric traits with SVR provided the lowest MAPE in this dataset. Deep feature performance was model-dependent and did not exceed the biometric-SVR configuration under the evaluated cattle-level cross-validation design.

## 4. Discussion

This study developed an integrated, multi-stage pipeline for cattle body segmentation and body weight estimation from overhead depth imagery. Accurate body weight monitoring is essential in beef production because growth, nutritional management, and reproductive performance are strongly associated with live weight, particularly in developing heifers [1]. However, conventional approaches such as scales and weight tapes require animal handling, labor, and dedicated infrastructure [2,3]. The proposed framework addresses these limitations by combining real-time segmentation, automated feature extraction, and regression-based weight prediction. Relative to prior image-based livestock phenotyping studies [7,8,28,29,30,31], this work provides an integrated overhead depth-imaging pipeline evaluated under animal-disjoint validation.

From a practical deployment perspective, the proposed system uses a single overhead Intel RealSense D435 RGB-D camera rather than a multi-camera system, which may reduce sensor cost, installation complexity, and calibration requirements. Nevertheless, large-scale commercial deployment would require a complete economic evaluation that includes the RGB-D camera, mounting and protective housing, local computing hardware or embedded processor, installation, calibration, maintenance, and integration with farm management software. Therefore, although the hardware configuration is relatively simple, future work should evaluate the total cost of ownership, labor savings, return on investment, and long-term robustness under commercial beef production conditions.

The segmentation results demonstrate that the proposed LAED + GCT architecture provides a favorable balance between accuracy and computational efficiency. The model achieved 96.91% mDice and 94.22% mIoU while operating at 33.08 FPS with 7.03 M parameters and 117.41 GFLOPs. Compared with U-Net, SAM2-U-Net, and Mask R-CNN, LAED + GCT achieved higher observed segmentation performance while requiring fewer parameters and lower computational cost. Paired MaxT analyses confirmed significant improvements over CBAM and DualAttn attention variants, while differences relative to no attention and ECA were not significant in our data. The ablation results further indicated that the multi-scale dilated bottleneck was the main architectural contributor, whereas the boundary head provided a smaller, non-significant improvement under the present dataset. The statistical analysis was conducted at the animal level, and the reported pairwise *p*-values were MaxT-adjusted permutation *p*-values that accounted for multiple paired comparisons within each metric.

The body weight estimation results indicate that performance depended on both feature representation and regression method. The best overall result was obtained using automatically derived biometric traits with SVR, which achieved MAPE = 6.75%, pooled $R^{2}$ = 0.68, MAE = 23.92 kg, and RMSE = 31.79 kg. This suggests that geometric descriptors extracted from segmented depth images captured relevant body-size information for weight prediction under the evaluated cattle-level cross-validation design.

The signed-error analysis provided additional information beyond MAE and RMSE. For the biometric-SVR model, the mean signed error was −1.04 kg when the signed error was defined as predicted minus observed body weight. Thus, the model showed slight average underestimation. However, the bootstrap 95% confidence interval for bias ranged from −9.63 to 7.41 kg and included zero. This indicates that the model did not show strong evidence of systematic overestimation or underestimation in the pooled out-of-fold predictions. The signed-error standard deviation was 33.85 kg, showing that individual-animal errors remained variable even though the average bias was small.

Deep feature representations also produced useful predictions, but their performance varied across backbones and regression algorithms. Among FCNN models trained independently within each cattle-level fold, ResNet50 features provided the best FCNN result, with a MAPE of 7.76%, a pooled $R^{2}$ of 0.56, an MAE of 27.60 kg, and an RMSE of 37.07 kg. FCNN results were competitive with several RF and SVR configurations, but did not exceed the best biometric-SVR model in this dataset. The relative advantage of SVR with biometric traits may be associated with the small animal-level sample size, because lower-dimensional biometric features can generalize more effectively than high-dimensional deep embeddings when the number of training animals is limited.

Comparison with previous studies further supports the competitiveness of the proposed method. With respect to MAPE, the best result obtained in this study was 6.75%, which is higher than the 3.13% reported by Kamchen et al. [29], but lower than the 8.4% reported by Ruchay et al. [7] and the approximately 13–17% reported by Rozendo et al. [31]. Because $R^{2}$ is also reported in the present study, comparison can be made with prior work using the same metric. The best model here achieved a pooled $R^{2}$ of 0.68, which is close to the $R^{2}=0.70$ reported by Miller et al. [28], near the lower end of the $R^{2}\;=\;0.69$–0.84 range reported by Gomes et al. [8], and lower than the $R^{2}=0.98$ reported by Xu et al. [30]. These comparisons should be interpreted cautiously because they involve different breeds, sample sizes, camera viewpoints, imaging modalities, preprocessing strategies, and evaluation protocols.

Recent studies provide additional context for the present results. Ruchay et al. [10] used three Kinect v2 RGB-D cameras and YOLOv8n-derived movement-speed features, achieving $R^{2}=0.845$, MAE = 18.01 kg, and MAPE = 4.38% on 199 cattle. In comparison, the present single-overhead-camera pipeline achieved $R^{2}=0.68$, MAE = 23.92 kg, RMSE = 31.79 kg, and MAPE = 6.75%, while also providing pixel-wise body segmentation through a lightweight real-time model. Nilchuen et al. [11] used smartphone-based side-view YOLOv11m detection and two linear measurements for Brahman cattle, achieving MAE = 43.44 kg and MAPE = 8.91% on 523 cows; the lower MAPE in the present study may reflect differences in imaging modality, view angle, feature extraction, and study population, while their approach offers lower-cost mobile accessibility. Menezes et al. [12] is the most directly comparable recent study because it also used top-down RealSense D435 videos. Their best body weight model achieved $R^{2}=0.95$ and RMSE = 24.2 kg on 94 beef-on-dairy cattle over six months. Their study reported stronger body-weight prediction performance and provided longitudinal evidence for growth-stage generalization. The present study contributes a dedicated lightweight semantic segmentation architecture with boundary-aware training, attention-module comparison, ablation analysis, and computational benchmarking, which are important because robust cattle body segmentation is a prerequisite for reliable automated phenotyping from overhead depth images.

An additional strength of this work is the explicit separation of animals between training and testing. In livestock imaging datasets, multiple frames from the same animal can lead to inflated performance estimates if splitting is performed at the frame level rather than at the animal level. By evaluating the models on previously unseen cattle, the present study provides a more realistic estimate of internal generalization performance. For body weight prediction, the 20 outer test folds were fixed before modeling and were used only for final out-of-fold evaluation. For SVR, hyperparameter tuning was restricted to outer-training cattle and did not use the three held-out cattle in the corresponding outer test fold. For FCNN models, each fold was trained from an independent initialization with no transfer of model weights, optimizer states, fitted feature scalers, dimensionality-reduction transformations, target-standardization parameters, validation information, or other training information across folds. However, several limitations should be considered when interpreting these results. Only a single manually selected frame per animal was annotated for segmentation, which may not fully capture the variability observed in continuous video streams, including differences in posture, partial occlusion, movement artifacts, and noisy depth measurements. In addition, the relatively small dataset size may limit statistical power and reduce the generalizability of the learned representations, particularly for high-dimensional deep features. Data collection was also conducted under controlled conditions using a single-animal lane, and the study population represented a relatively narrow range of ages and management environments. Furthermore, the biometric volume descriptor used in this study was an uncalibrated proxy rather than a direct volumetric measurement, and the current framework did not incorporate temporal, locomotion, or behavioral information that may further improve prediction performance. Despite these limitations, the present study demonstrates the feasibility of the LAED + GCT approach for cattle body weight prediction. These results provide a foundation for developing more scalable and non-invasive phenotyping approaches in commercial production systems.

## 5. Conclusions

The proposed LAED + GCT segmentation model achieved high accuracy for cattle body segmentation from overhead depth imagery, reaching 96.91% mDice and 94.22% mIoU while maintaining real-time processing at 33.08 frames per second. These segmentation outputs enabled body weight estimation from non-invasive image-derived features. For body weight estimation, the best primary-metric regression pipeline used automatically derived biometric traits with SVR, achieving MAPE = 6.75%, pooled $R^{2}$ = 0.68, MAE = 23.92 kg, and RMSE = 31.79 kg. The mean signed prediction bias for this model was −1.04 kg, using predicted minus observed body weight, with a bootstrap 95% confidence interval of −9.63 to 7.41 kg. Among FCNN models trained independently within each cattle-level fold, the best result used ResNet50 features, with MAPE = 7.76%, pooled $R^{2}$ = 0.56, MAE = 27.60 kg, and RMSE = 37.07 kg. Together, these results demonstrate that depth-based computer vision can provide reliable body measurements and weight predictions from non-invasive image data under the evaluation protocol used in this study.

Our findings highlight the potential of automated depth imaging systems to support practical body weight monitoring in beef cattle operations. By reducing the need for repeated animal handling and conventional weighing procedures, this approach could enable more frequent phenotypic data collection and improve decision-making related to growth monitoring, nutrition, and reproductive management. However, larger external validation, prospective testing, economic evaluation, and edge-device benchmarking may be needed before broad commercial deployment.

## Figures and Tables

**Table 1:** Animal-level segmentation performance for LAED attention variants. Values are reported as mean ± standard deviation [95% bootstrap confidence interval]. Confidence intervals are animal-level nonparametric percentile bootstrap intervals based on 20,000 bootstrap resamples.

Proposed Models	mDice (%)	mIoU (%)
LAED (No Attention)	96.58 ± 1.80 [96.08, 96.98]	93.62 ± 3.19 [92.75, 94.33]
LAED + DualAttn	96.46 ± 1.69 [96.01, 96.85]	93.39 ± 3.06 [92.57, 94.09]
LAED + ECA	96.41 ± 1.96 [95.88, 96.86]	93.32 ± 3.50 [92.36, 94.13]
LAED + CBAM	96.39 ± 1.51 [95.99, 96.76]	93.26 ± 2.77 [92.53, 93.92]
LAED + GCT	96.91 ± 1.29 [96.56, 97.21]	94.22 ± 2.37 [93.58, 94.77]

**Table 2:** Paired MaxT permutation tests comparing LAED + GCT with attention variants. Differences are reported in percentage points. *p*-values are two-sided studentized MaxT-adjusted paired permutation *p*-values computed from animal-level paired differences and adjusted within each metric across the four pairwise comparisons.

Comparator	ΔDice	MaxT-Adjusted *p*	ΔIoU	MaxT-Adjusted *p*
No Attention	+0.33	0.262	+0.60	0.261
ECA	+0.50	0.161	+0.90	0.151
CBAM	+0.52	0.009	+0.96	0.008
DualAttn	+0.45	0.030	+0.83	0.030

**Table 3:** Segmentation performance and computational efficiency across baseline segmentation models.

Segmentation Models	mDice	mIoU	Params (M)	GFLOPs	Latency (ms)	FPS
U-Net	95.23	91.05	17.26	495.74	42.14	23.73
SAM2-U-Net	95.51	91.99	216.53	256.48	49.76	20.10
Mask R-CNN	95.62	91.63	43.98	507.51	118.67	8.43
LAED + GCT	96.91	94.22	7.03	117.41	30.23	33.08

**Table 4:** Ablation results for LAED + GCT. Confidence intervals are reported in brackets. Confidence intervals are animal-level nonparametric percentile bootstrap intervals based on 20,000 bootstrap resamples. *p*-values are two-sided studentized MaxT-adjusted paired permutation *p*-values computed from animal-level paired differences.

Model	mDice (%)	mIoU (%)	Adj. *p* Dice	Adj. *p* IoU
Full LAED + GCT	96.91 [96.56, 97.20]	94.22 [93.60, 94.77]	–	–
w/o boundary head	96.79 [96.35, 97.17]	94.01 [93.19, 94.70]	0.446	0.468
w/o boundary and dilated	96.31 [95.82, 96.73]	93.12 [92.24, 93.88]	0.004	0.003
w/o dilated bottleneck	95.91 [95.39, 96.37]	92.37 [91.42, 93.21]	<0.001	<0.001

**Table 5:** Body weight estimation performance across feature sets and regression models. MAPE is reported in %; MAE and RMSE are reported in kg. Metrics were computed from pooled out-of-fold predictions. FCNN results were obtained using independent cattle-level cross-validation with fold-specific initialization and no transfer of model weights or optimizer states across folds. Bold values indicate the strongest result within each feature set for each metric.

Feature Set	Model	MAPE (%)	$R^{2}$	MAE (kg)	RMSE (kg)
Biometric	SVR	**6.75**	**0.68**	**23.92**	**31.79**
Biometric	RF	7.33	0.56	26.32	36.94
Biometric	FCNN	8.42	0.53	29.24	38.43
DenseNet121	SVR	**7.59**	**0.64**	**27.16**	**33.47**
DenseNet121	RF	8.32	0.55	29.25	37.53
DenseNet121	FCNN	8.82	0.51	31.31	39.13
ResNet50	SVR	9.42	0.40	33.08	42.97
ResNet50	RF	8.50	0.52	29.57	38.54
ResNet50	FCNN	**7.76**	**0.56**	**27.60**	**37.07**
ResNeXt50	SVR	9.61	0.44	33.00	41.61
ResNeXt50	RF	8.65	0.51	30.20	39.23
ResNeXt50	FCNN	**8.38**	**0.52**	**29.64**	**38.67**

## Data Availability

The raw data supporting the conclusions of this article will be made available by the authors on request.
